# Comparative analysis of IgG and IgG subclasses against *Plasmodium falciparum* MSP-1_19_ in children from five contrasting bioecological zones of Cameroon

**DOI:** 10.1186/s12936-019-2654-9

**Published:** 2019-01-22

**Authors:** Tebit Emmanuel Kwenti, Tufon Anthony Kukwah, Tayong Dizzle Bita Kwenti, Babila Raymond Nyassa, Meriki Henry Dilonga, George Enow-Orock, Nicholas Tendongfor, Nota Damian Anong, Samuel Wanji, Longdoh Anna Njunda, Theresa Nkuo-Akenji

**Affiliations:** 1 Department of Medical Laboratory Sciences, Faculty of Health Science, University of Buea, P.O. Box 23, Buea, Southwest Region Cameroon; 2 Regional Hospital of Buea, P.O. Box 32, Buea, Southwest Region Cameroon; 3 Department of Public Health and Hygiene, Faculty of Health Science, University of Buea, P.O. Box 23, Buea, Southwest Region Cameroon; 4 Department of Microbiology and Parasitology, Faculty of Science, University of Buea, P.O. Box 63, Buea, Southwest Region Cameroon; 5 Department of Biomedical Science, Faculty of Health Science, University of Buea, P.O Box 23, Buea, Southwest Region Cameroon; 6 Parasites and Vector Biology Research Unit, Department of Microbiology and Parasitology, University of Buea, Buea, Cameroon; 7 Department of Biological Science, Faculty of Science, University of Bamenda, Bamenda, North West Region Cameroon

**Keywords:** MSP-1, Malaria immune responses, IgG subclasses, Children, ELISA, Bioecological Strata, Cameroon

## Abstract

**Background:**

Studies reporting the natural immune responses against malaria in children from different geographical settings in endemic areas are not readily available. This study was aimed at comparing the immune responses against *Plasmodium falciparum* MSP-1_19_ antigen in children from five contrasting bioecological zones in Cameroon.

**Methods:**

In a cross-sectional survey, children between 2 and 15 years, were enrolled from five ecological strata including the south Cameroonian equatorial forest, sudano-sahelian, high inland plateau, high western plateau, and the coastal strata. The children were screened for clinical malaria (defined by malaria parasitaemia ≥ 5000 parasites/µl plus axillary temperature ≥ 37.5 °C). Their antibody responses were measured against *P. falciparum* MSP-1_19_ antigen using standard ELISA technique.

**Results:**

In all, 415 children comprising 217 (52.3%) males participated. Total IgG and IgG1–IgG4 titres were observed to increase with age in all the strata except in the sudano-sahelian and high inland plateau strata. Total IgG and IgG1–IgG4 titres were significantly higher in the coastal strata and lowest in the high inland plateau (for IgG1 and IgG2) and sudano-sahelian strata (for IgG3 and IgG4). Titres of the cytophilic antibodies (IgG1 and IgG3) were significantly higher than the non-cytophilic antibodies (IgG2 and IgG4) in all the strata except in the sudano-sahelian and high inland plateau strata. Total IgG and IgG subclass titres were significantly higher in children positive for clinical malaria compared to negative children in all study sites except in the high western plateau and coastal (for IgG1 and IgG3), and the sudano-sahelian strata (for all antibodies). Furthermore, a significant positive correlation was observed between parasite density and IgG2 or IgG4 titres in all study sites except in the south Cameroonian equatorial forest and sudano-sahelian strata.

**Conclusions:**

This study showed that antibody responses against MSP-1_19_ vary considerably in children from the different bioecological strata in Cameroon and could be linked to the differential exposure to malaria in the different strata. Furthermore, the rate of antibody acquisition was not observed to increase in an age-dependent manner in low transmission settings.

**Electronic supplementary material:**

The online version of this article (10.1186/s12936-019-2654-9) contains supplementary material, which is available to authorized users.

## Background

Malaria, a disease caused by a parasitic protozoan of the genus *Plasmodium*, remains a major public health challenge in sub-Saharan Africa (SSA). In 2017, there were an estimated 219 million cases and 435,000 deaths attributed to malaria worldwide [1]. Approximately 80% of cases and death attributed to malaria occurs in SSA [1]. Although there has been a decline in the number of new cases and death attributed to malaria recently, the disease is still a major cause of morbidity and mortality in children in SSA, claiming the life of a child every 2 min [2].

Malaria in Cameroon is a major cause of morbidity and mortality especially in children [3]. In the country, malaria accounts for 48% of all hospital admissions, 30% of morbidity and 67% of childhood mortality per year [4, 5]. *Plasmodium falciparum* is the predominant species causing malaria in Cameroon [6] and the entire population of over 22 million is at risk of malaria [7].

The epidemiology of malaria in Cameroon is complex and has been described as “Africa in miniature” [8], because Cameroon has all the bioecological strata of malaria present in Africa. There are six bioecological strata namely: the sudano-sahelian, high inland plateau, savannah-forest transmission, south Cameroonian equatorial forest, high western plateau, and the coastal strata [9]. These strata differ substantially in terms of their geo-ecological characteristics, transmission pattern and endemicity levels as well as in terms of the main vectors transmitting malaria parasites [9].

In malaria, immunoglobulin G (IgG) antibodies are known to play a vital role in combating infection by reducing parasitaemia and clinical symptoms [10–13]. Among the IgG subclasses, the cytophilic antibodies (IgG1 and IgG3) have been considered the most important as they are capable of mediating the activation of leukocytes via binding to FcγRI and FcγRIII. In malaria-endemic areas, the predominance of IgG1 and IgG3 is associated with lower risks of malaria-related complications [14–17]. On the contrary, the non-cytophilic antibodies especially IgG4 are known to be pathogenic and their presence correlates with the severity of malaria [15, 18, 19]. However, a balance between the cytophilic (IgG1 and IgG3) and non-cytophilic (IgG2 and IgG4) antibodies is required for the development of effective immunity against malaria [20–22].

This study evaluated the humoral (IgG) immune responses against merozoite surface protein 1 (MSP-1). MSP-1 is one of the best-characterized proteins in *Plasmodium* spp. It is the most abundant merozoite surface protein and is thought to be involved in the initial attachment of the merozoite to the erythrocyte surface [23]. The 19 kDa C-terminal fragment of MSP-1 (MSP-1_19_) has been recognized as the target of IgG-based protective immunity [24] and is a promising vaccine candidate [25]. MSP-1_19_ was selected over the other MSP-1 molecules because of the fine specificity of MSP-1_19_ specific antibodies [26, 27] coupled to its role in protecting against clinical malaria. Joos et al. [28] showed that MSP-1_19_ specific antibodies are potent inducers of neutrophil antibody-dependent respiratory burst (ADRB), which correlates with acquired clinical protection. Furthermore, studies have demonstrated the role of the cytophilic antibodies in ADRB [29, 30].

Previous studies have shown that malaria transmission in Cameroon decreases steadily northward, from the coastal (C) strata in the south to the SS strata in the north [3, 6], while the immune responses against malaria decrease in this direction [31]. No study has compared the IgG subclass responses against malaria parasites in different geographical areas in Cameroon; the study by Kwenti et al. [31] measured only total IgG responses. Therefore, a gap still exists in our understanding of the IgG subclass responses in children across the different bioecological strata of malaria, as well as the identity of the IgG subclasses which are protective against clinical malaria. In this study, children (2–15 years) were enrolled from five bioecological strata and their immune responses (total IgG and IgG1–4) against MSP-1_19_ recombinant antigens were compared. The aim was to generate data that will improve understanding of the immune response against malaria in different geographical zones and may be useful in designing future trials for testing of malaria vaccine candidates.

## Methods

### Study area

**Fig. 1:**
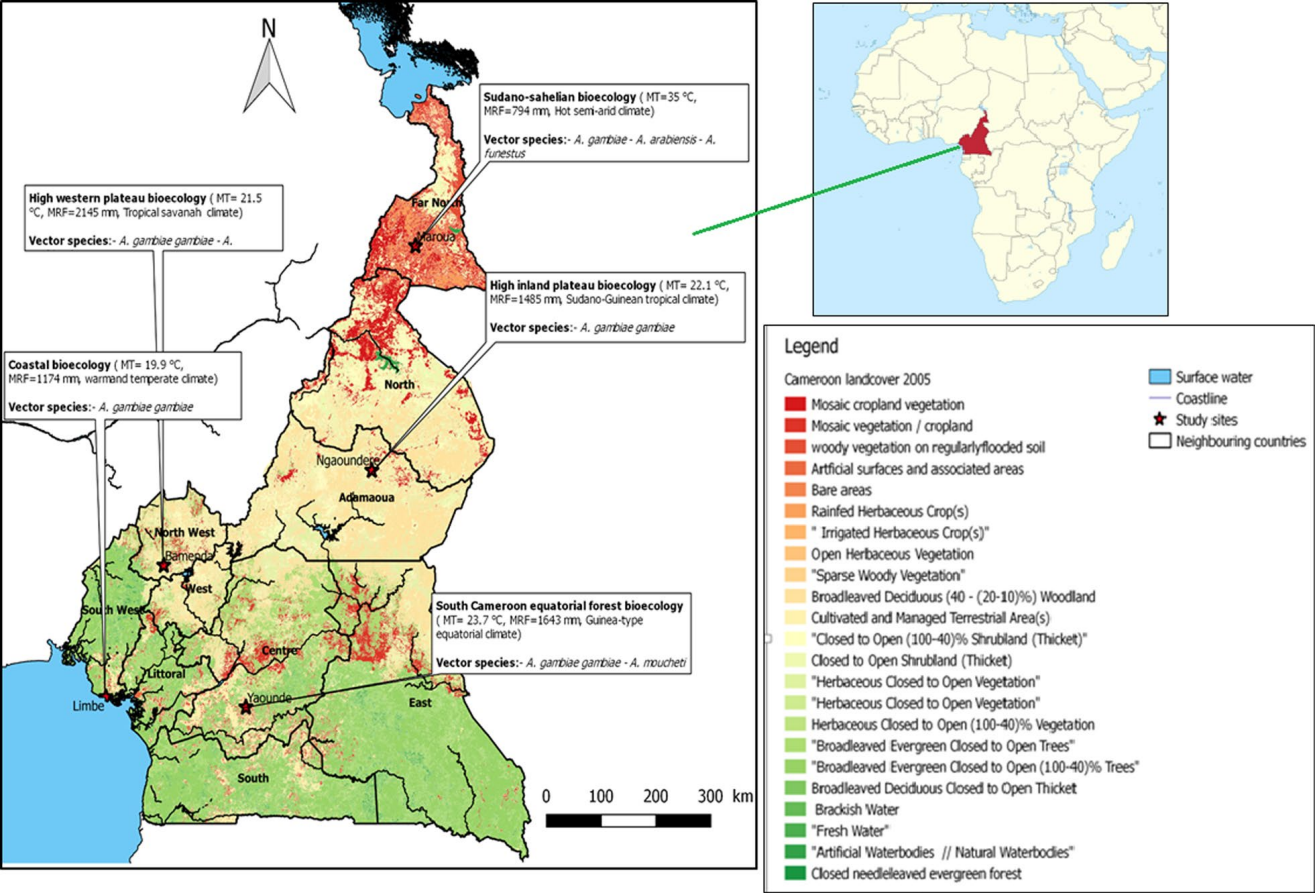
Map showing the five study sites. The characteristics of the five bioecological strata are delineated

Five out of the six bioecological strata of malaria were randomly selected and participants were enrolled from five study sites, one for each strata including: Maroua in the sudano-sahelian strata, Ngaoundere in the high inland plateau strata, Yaoundé in the south Cameroonian equatorial forest strata, Bamenda in the high western plateau strata and Limbe in the coastal strata (Fig. 1).

The sudano-sahelian (SS) strata constitute the dry savannah zones and the steppes to the north of the country. It lies between latitudes 13° N and 8° N. The SS strata is characterized by a hot semi-arid climate and the vegetation is of the sahelian type. There is not much rainfall in this zone; the mean annual rainfall is 794 mm (ranging from 0 to 245 mm). The average temperature is 35 °C (ranging from 28 to 45 °C). Malaria in the SS strata is unstable with a risk of an epidemic and severe clinical forms that affect all ages [9]. *Plasmodium falciparum* is the main species causing malaria and its main vector is *Anopheles gambiae* [9]. In Maroua where the study was conducted, the entomological inoculation rates (EIR) is reported as 0.16 bites per man per night during the rainy season [32].

The high inland plateau (HIP) strata is found in the very heart of Cameroon, between latitudes 8° N and 6° N. It is characterized by the Guinea-Savannah type vegetation and the Sudano-Guinean tropical climate is tempered by the altitude (1100 m on average). The average temperature is 22.1 °C and mean annual rainfall is 1485 mm (ranging from 0 to 280 mm). Malaria in the HIP strata is tropical and stable with seasonal outbreaks, caused mainly by *P. falciparum*. Data on the entomological inoculation rate was not readily available for Ngaoundere, where this study was conducted.

The south Cameroonian equatorial forest (SCEF) strata lie between latitudes 5° N and 2° N. It is characterized by the Guinea-type equatorial climate and the vegetation is comprised of the equatorial evergreen forest. The average temperature is 23.7 °C (ranging between 22.6 °C and 24.6 °C) and the mean annual rainfall is 1643 mm. Malaria in the SCEF strata is holoendemic, caused mainly by *P. falciparum* and *An. gambiae* is the principal vector. In Yaoundé, where this study was conducted, the entomological inoculation rates is reported as 6 bites per man per month during the rainy season [33].

The coastal (C) strata corresponds to the only coastal region in Cameroon. It ranges from Campo to Mamfe. Its altitude is inferior to 300 mm and this is a vegetable cul-de-sac often swallowed up by the monsoon. The climate is warm and temperate. The average temperature is 19.9 °C (ranging from 15.8 to 32.8 °C) and the mean annual rainfall is 1174 mm (ranging from 27 to 617 mm). In this zone, malaria transmission is highest in the country, caused mainly by *P. falciparum* and *An. gambiae* is the main vector [9]. In Limbe, where the current study was conducted, the entomological inoculation rates is reported as 0.45 bites per man per night during the rainy season [34].

The high western plateau (HWP) strata is a polygon-shaped region stretching 300 km by 200 km. It is characterized by the tropical savanna climate and Sudan savanna forms the dominant vegetation. The mean temperature is 21.5 °C (ranging from 20.1 to 23.0 °C) and the mean annual rainfall is 2145 mm (ranging from 9 to 383 mm). Malaria transmission in the HWP strata is stable, occurring all year long. *P. falciparum* is the main species and *An. gambiae* is the main vector. Data on the entomological inoculation rate was not readily available for Bamenda, where this study was conducted.

### Study design and duration

This was a cross-sectional comparative survey conducted on children from five bioecological strata of malaria in Cameroon. Data were collected between April and July 2015 in all the strata (to coincide with the rainy season during which transmission is higher).

### Study population

Children (2–15 years) of both sexes were randomly selected from the community in the different study sites. Eligible participants here were those who had not been on any anti-malarial drug for at least a week prior to enrolment.

### Sampling size determination

Using the effect size of 0.18 deduced from the study by Nebie et al. [13], and using power (1 − β) = 0.8, α = 0.05, and the ANOVA function in G*Power with five groups, a sample size of 75 participants per study site was obtained, giving a grand total of at least 375 (5 × 75).

### Sampling technique

In order to ensure that the data obtained is a true representation of the study site, a multistage sampling technique was used to sample apparently healthy children (2–15 years) from the community by firstly, randomly selecting three neighbourhoods, and secondly, randomly selecting 135 houses within the neighbourhoods and one child per house who met the inclusion criteria, was selected to obtain the required sample size. This was done in all the study sites.

### Sample collection

From every participant, about 3 ml of venous blood was collected into EDTA anticoagulated tube and non-coagulated (dry) tube following antiseptic techniques. Sample from the EDTA tube was used in the preparation of blood films and performing complete blood count (CBC), while sera from the dry tube were used to perform ELISA.

### Detection and quantification of the malaria parasite

The prepared blood films were air-dried and stained with 10% Giemsa (1 in 20 dilutions) for 25–30 min [35]. The blood films were read by two expert microscopists who were blinded from each other’s result. In the case of any discrepancy with the results obtained by the two microscopists, a third was brought in and the result he gave was considered as final. Two hundred fields were screened for malaria parasite using the 100× (oil immersion) objective and where parasites were detected, the parasites were counted until 500 WBC was reached. The slides were declared negative only after counting 2500 WBC. The parasite density was estimated by dividing the number of parasites counted by 500 WBC and then multiplied by the actual WBC count (obtained from the CBC results) of the participant to give numbers in parasite per μl of blood [36].

### Measurement of total IgG and IgG subclasses

The total IgG and IgG subclass responses were measured against PfMSP-1_19_ antigen (expressed in *Escherichia coli* and purified to 95%) using a standard ELISA technique as earlier described [31]. Briefly, 96 well microtitre plates (Nunc Maxisorb™, Denmark) were coated overnight at 4 °C with 100 µl (1×PBS) of the recombinant protein solution (PfMSP-1_19_) at a final concentration of 1 µg/ml. The unbound antigens were removed by washing 3 times with 0.1% Tween/PBS washing buffer. After washing, 150 µl/well of 3% skimmed milk powder in Tween/PBS was used to block the wells for 8 h at 4 °C, followed by plate washing as described above. After the second wash, sera samples (diluted 1 in 100) were added in duplicate, along with positive control serum (a pool of sera from eight adults in Muyuka with lifelong exposure to malaria) and 10 negative control sera from non-exposed German adults (provided by Andreas Latz). The plates were then incubated at 37 °C for 1 h before washing as described above. Peroxidase-conjugated goat anti-human IgG (Caltag) for IgG (1/40000) and mouse anti-human IgG1, IgG2, IgG3 and IgG4 (Arigo Biolaboratories Corp., Taiwan) for IgG1 (1/6000), IgG2 (1/4000), IgG3 (1/6000) and IgG4 (1/5000) were added (100 µl/well) and incubated for 30 min at room temperature (RT). Afterward, 100 µl/well TMB substrate was added and incubated for 15 min in the dark. The reaction was then stopped by adding 100 µl/well of 0.2 M sulphuric acid (H_2_SO_4_) and absorbance was read at 450 nm with an ELISA plate reader, BioTek^®^ ELx800TM (BioTek Instruments, Inc., USA). A standard curve derived from serial dilution (1:200, 1:400, 1:800, 1:1600, and 1:3200) of positive control sera for all test plates, was used to convert antibody responses to arbitrary units (AU), with the absorbance of the lowest dilution corresponding to 100 AU [31].

### Data analysis

Frequency tables and charts were used to present data. Age was grouped into three categories: 1 (< 5 years), 2 (5–9 years), 3 (≥ 10 years). The statistical tests performed included the Student’s t-test and ANOVA for the comparison of group mean, multiple linear regression analysis was also used to compare antibody concentrations between groups adjusting for possible confounding (likely confounders in this study included age, gender and study sites). The data was checked for clustering using the K-means cluster and the Hierarchical cluster methods and clustering was insignificant. All continuous variables were standardized prior to statistical analysis by logarithmic transformation. Statistical significance was set at p ≤ 0.05. For the comparison of IgG antibody responses, clinical malaria was defined as malaria parasitaemia (≥ 5000 parasites/µl) plus fever (axillary temperature ≥ 37.5 °C). Data collected were entered into an Excel spreadsheet and analysed using the Stata^®^ version 12.1 software (StataCorp LP, Texas, USA) and SPSS statistical software version 20 (IBM, USA).

## Results

### Characteristics of the study population

In all, 415 children consented and took part in the study. There were 83, 82, 82, 85 and 83 participants from Bamenda, Limbe, Maroua, Ngaoundere, and Yaoundé, respectively (Table 1). Overall, there were 217 (52.3%) males and 198 (47.7%) females (Table 1). The mean (± SD) ages of the participants was 6.1 (± 3.6). Between the different study sites, the participants did not differ in terms of their gender (χ^2^ = 1.732, p = 0.785) and their mean ages (p = 0.439).

**Table 1:** Age and gender distribution of the study participants

Bioecological strata	Study site		Age category (years)			Total
			< 5 n (%)	5–9 n (%)	≥ 10 n (%)	
HWP	Bamenda	Gender				
		F	16 (43.2)	10 (27.0)	11 (29.7)	37
		M	19 (41.3)	16 (34.8)	11 (23.9)	46
		Total	35 (42.2)	26 (31.3)	22 (26.5)	83
C	Limbe	Gender				
		F	18 (40.9)	16 (36.4)	10 (22.7)	44
		M	22 (57.9)	11 (28.9)	5 (13.2)	38
		Total	40 (48.8)	27 (32.9)	15 (18.3)	82
SS	Maroua	Gender				
		F	18 (48.6)	13 (35.1)	6 (16.2)	37
		M	24 (53.3)	16 (35.6)	5 (11.1)	45
		Total	42 (51.2)	29 (35.4)	11 (13.4)	82
HIP	Ngaoundere	Gender				
		F	24 (60.0)	11 (27.5)	5 (12.5)	40
		M	25 (55.6)	12 (26.7)	8 (17.8)	45
		Total	49 (57.7)	23 (27.1)	13 (15.3)	85
SCEF	Yaoundé	Gender				
		F	11 (27.5)	17 (42.5)	12 (30.0)	40
		M	12 (27.9)	12 (27.9)	19 (44.2)	43
		Total	23 (27.7)	29 (34.9)	31 (37.4)	83
Total		Gender				
		F	87 (43.9)	67 (33.8)	44 (22.2)	198
		M	102 (47.0)	67 (30.9)	48 (22.1)	217
		Total	189 (45.5)	134 (32.3)	92 (22.2)	415

SCEF: south Cameroonian equatorial forest strata, SS: sudano-sahelian strata, HIP: high inland plateau strata, HWP: high western plateau strata, C: coastal strata

The overall prevalence of clinical malaria was 16.9% (70/415, 95% CI 13.4–20.8). Malaria prevalence was highest in Limbe (in the coastal strata) and lowest in Maroua (in the sudano-sahelian strata) (p < 0.0001). Malaria prevalence was highest in children between 5 and 9 years and lowest in children below 5 years (p = 0.004). No significant difference was observed between malaria prevalence and gender (p = 0.227) (Table 2). *Plasmodium falciparum* was the only species identified as the cause of clinical malaria. The geometric mean parasite density was 30,564.5 parasites/μl.

**Table 2:** Distribution of clinical malaria in the study population stratified according to study site, age, and gender

Parameter	n	Clinical malaria present n (%)	Univariate analysis	
			χ^2^	p-value
Ecological strata/study site				
HWP/Bamenda	83	14 (16.9)	24.005	< 0.0001
C/Limbe	82	25 (30.5)		
SCEF/Yaoundé	83	18 (21.7)		
SS/Maroua	82	3 (3.7)		
HIP/Ngaoundere	85	10 (11.8)		
Total	415	70 (16.9)		
Age (years)				
< 5	189	20 (10.6)	11.249	0.004
5–9	134	33 (24.6)		
≥ 10	92	17 (18.5)		
Total	415	70 (16.9)		
Gender				
Females	198	38 (19.2)	1.459	0.227
Males	217	32 (14.7)		
Total	415	70 (16.9)		

SCEF: south Cameroonian equatorial forest strata, SS: sudano-Sahelian strata, HIP: high inland plateau strata, HWP: high western plateau strata, C: coastal strata, χ^2^: Chi square

### Variation of IgG titres to MSP-1_19_ with respect to age

**Fig. 2:**
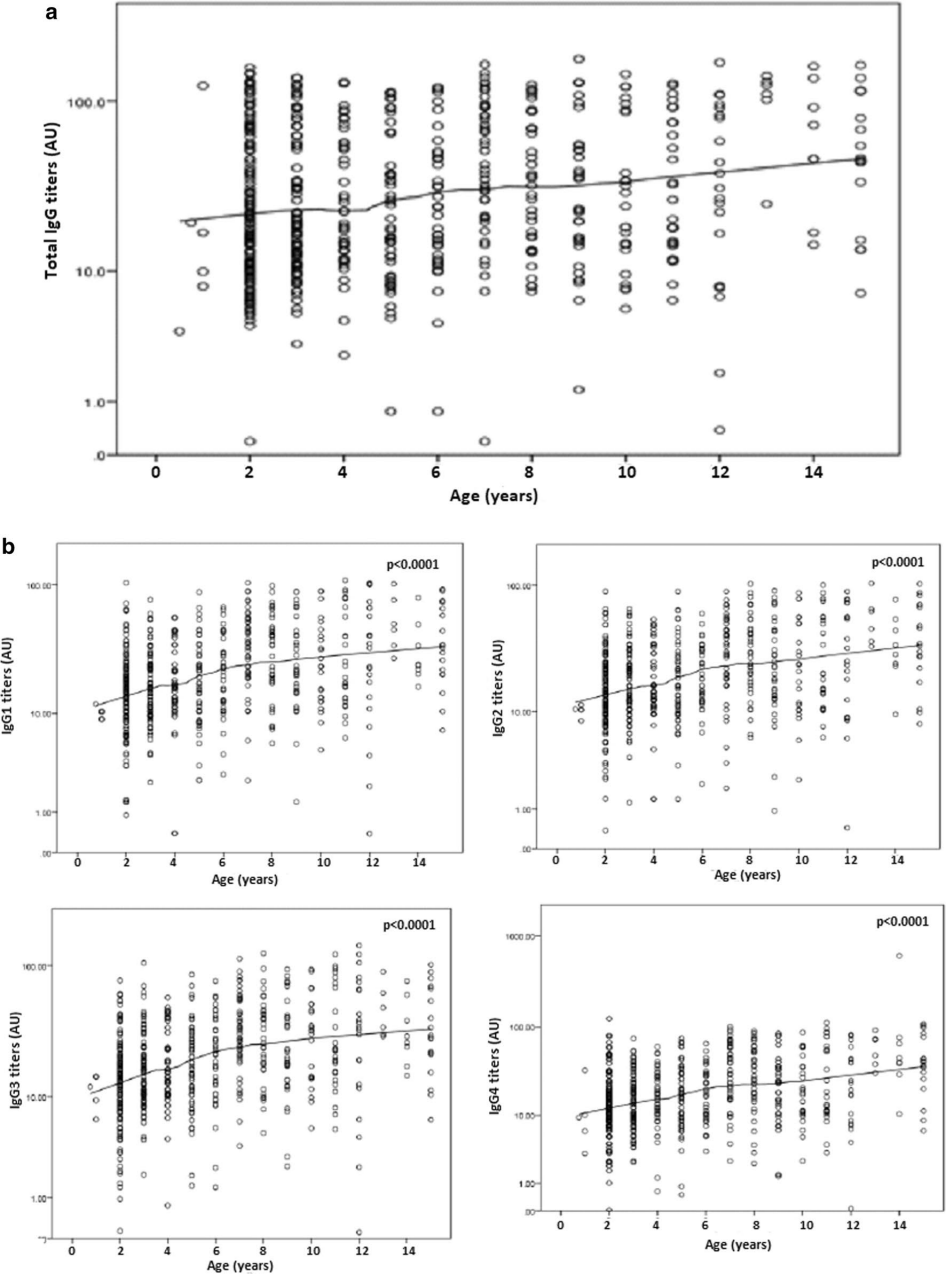
Overall variation of antibody levels to MSP-1_19_ with age in the study population. The line shows the LOESS smoothed estimate of the geometric mean. Evidence shows that total IgG (**a**) and IgG1–4 (**b**) levels increased with increasing age adjusting for gender and study site (p < 0.0001)

All the participants had detectable levels of IgG generally. Overall total IgG and IgG subclass levels were observed to increase with age (p < 0.0001) adjusting for gender and study site (Fig. 2). Total IgG antibody levels were observed to increase with age in all the study sites with the exception of Maroua and Ngaoundere (Additional file 1). Site-specific analysis revealed that IgG1, IgG2, IgG3, and IgG4 levels increased with age in Bamenda, Limbe, and Yaoundé, but only IgG1 and IgG2 increased with age in Maroua meanwhile none of the IgG subclass levels increased with age in Ngaoundere (Additional file 1).

### Variation of IgG titres to MSP-1_19_ with respect to gender

Globally, total IgG levels were observed to be independent of gender (p = 0.055) adjusting for age and study site. Although not significant, the total IgG levels were higher in females in all the study sites except in Limbe where it was higher in males. Site-specific analysis of the levels of the different IgG subclasses revealed no significant association with gender, except in Maroua where females were observed to have significantly higher titres of IgG2 (p = 0.030) and IgG4 (p = 0.016) adjusting for age and study site.

### Variation of IgG titres to MSP-1_19_ with respect to the study site

**Fig. 3:**
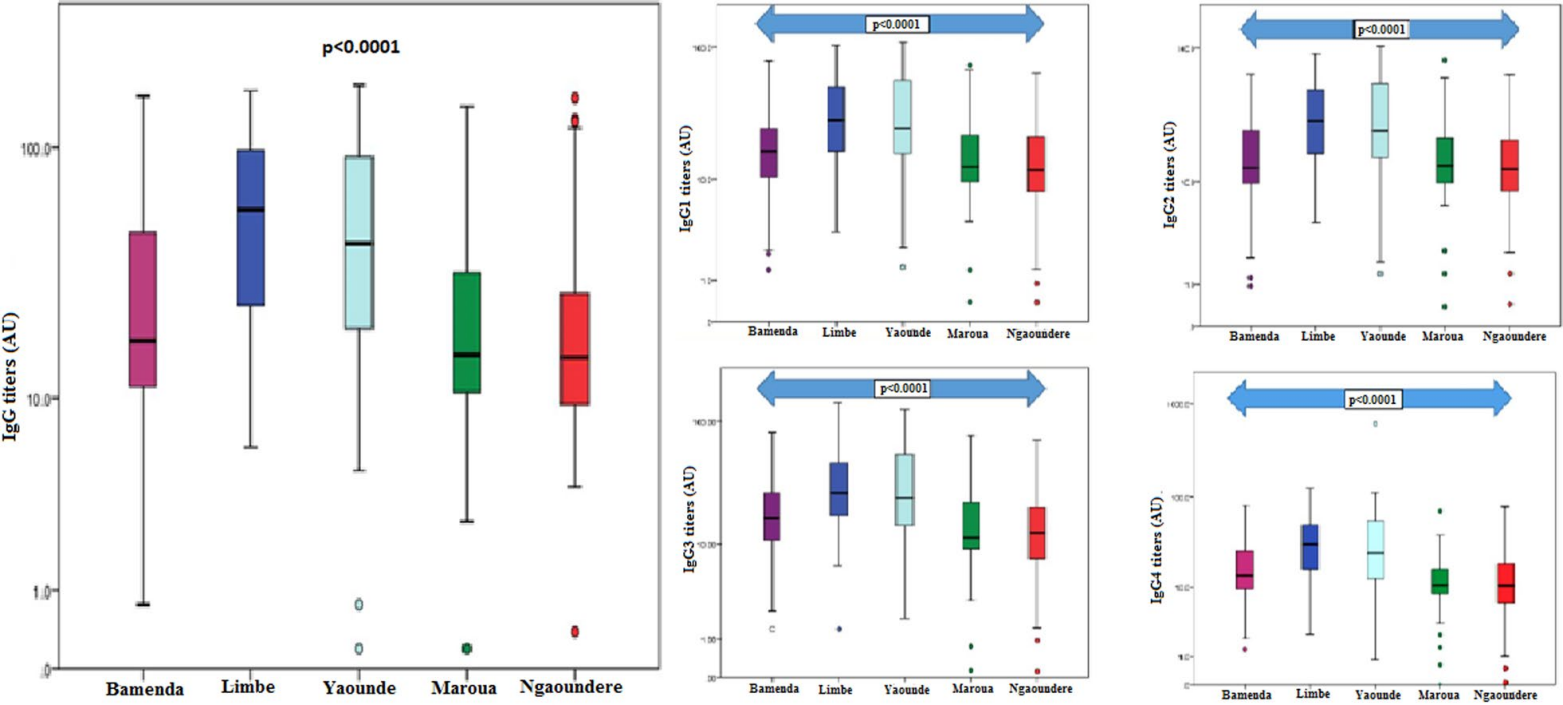
Variation of the total IgG antibody levels (log-transformed) to MSP-1_19_ with study site. There is evidence of significant association between total IgG, IgG subclass levels and study site

Total IgG levels were observed to vary with study site, being highest in Limbe and lowest in Ngaoundere (Fig. 3). Multiple linear regression analysis revealed this association to be significant adjusting for age and gender (p < 0.0001). IgG1 and IgG2 levels were highest in Limbe and lowest in Ngaoundere, meanwhile, IgG3 and IgG4 levels were highest in Limbe and lowest in Maroua (Fig. 3).

Site-specific analysis of the different IgG subclasses revealed that the cytophilic antibodies (IgG1 and IgG3) were consistently higher than the non-cytophilic antibodies (IgG2 and IgG4) in all the study sites except in Maroua and Ngaoundere (Table 3). Within site comparison of the different IgG subclasses revealed significant differences between IgG subclasses in Bamenda, Yaoundé, and Limbe but not in Maroua and Ngaoundere, adjusting for age and gender (Table 3).

**Table 3:** Comparison of the levels of the different IgG subclasses within the different study sites

Bioecological strata	Study site	IgG1	IgG2	IgG3	IgG4	IgG subtype pattern	p-value
HWP	Bamenda	1.8017 ± 0.3213	1.1537 ± 0.3213	2.2087 ± 0.3238	1.1843 ± 0.3299	IgG3 > IgG1 > IgG4 > IgG2	0.017
SCEF	Yaoundé	2.4315 ± 0.3722	1.4344 ± 0.3673	2.4139 ± 0.3769	1.3981 ± 0.437	IgG1 > IgG3 > IgG2 > IgG4	0.003
HIP	Ngaoundere	1.0869 ± 0.3791	1.1013 ± 0.3487	1.0632 ± 0.3988	1.0096 ± 0.4467	IgG2 > IgG1 > IgG3 > IgG4	0.274
SS	Maroua	1.1305 ± 0.3439	1.2379 ± 0.3532	1.1015 ± 0.3996	1.0139 ± 0.4510	IgG2 > IgG1 > IgG3 > IgG4	0.184
C	Limbe	2.4534 ± 0.2970	1.4371 ± 0.2862	2.4409 ± 0.3176	1.4311 ± 0.3259	IgG1 > IgG3 > IgG2 > IgG4	0.011
	Overall	2.2754 ± 0.3751	1.2657 ± 0.3663	2.2617 ± 0.3922	1.2283 ± 0.4332	IgG3 > IgG1 > IgG2 > IgG4	0.027

The figures represent mean antibody titres and their respective standard deviations. ANOVA was used to compare the mean antibody levels of the different IgG subclasses for each study site

SCEF: South Cameroonian equatorial forest strata, SS: sudano-sahelian strata, HIP: high inland plateau strata, HWP: high western plateau strata, C: coastal strata

### Comparison of IgG levels to MSP-1_19_ between children with and without clinical malaria

**Fig. 4:**
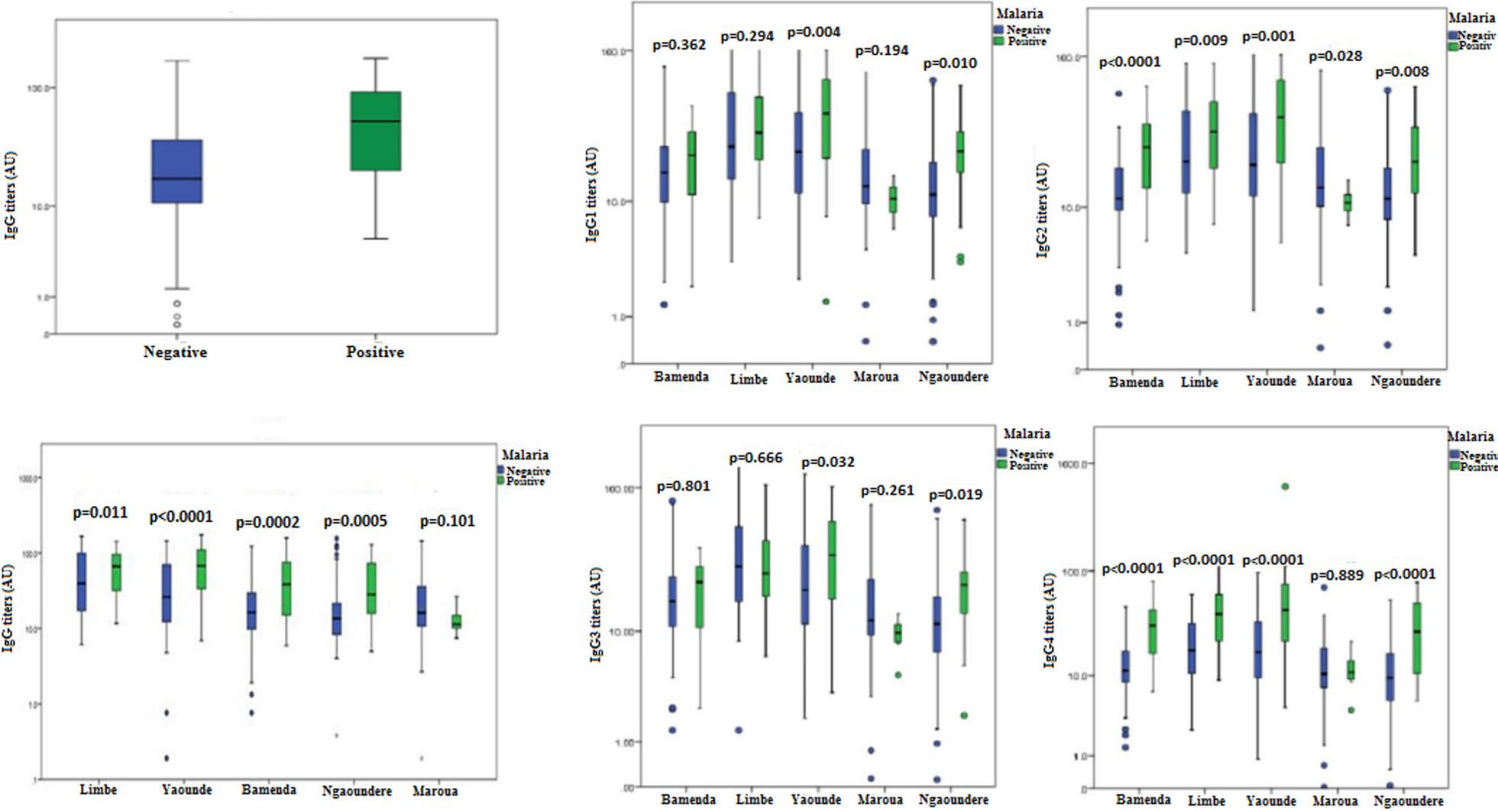
Variation of the total IgG and IgG1–4 levels (log-transformed) to MSP-1_19_ between children positive and negative for clinical malaria. It shows that antibody level was higher in positive children compared to negative children (p < 0.0001) adjusting for age, gender and study site

Overall, the total IgG antibody concentration was observed to be higher in children positive for clinical malaria compared to negative children (Fig. 4). Multiple linear regression analysis revealed this difference to be significant adjusting for age, gender, and study site (p < 0.0001). Total IgG levels were higher in children positive for clinical malaria in all the study sites with the exception of Maroua where total IgG levels were similar between children that were positive and negative for clinical malaria.

Site-specific analysis of the different IgG subclasses revealed no significant differences in the titres between children that were positive and negative for clinical malaria for IgG1 and IgG3 in Bamenda, Limbe, and Maroua, and IgG2 and IgG4 in Maroua (Fig. 4).

**Fig. 5:**
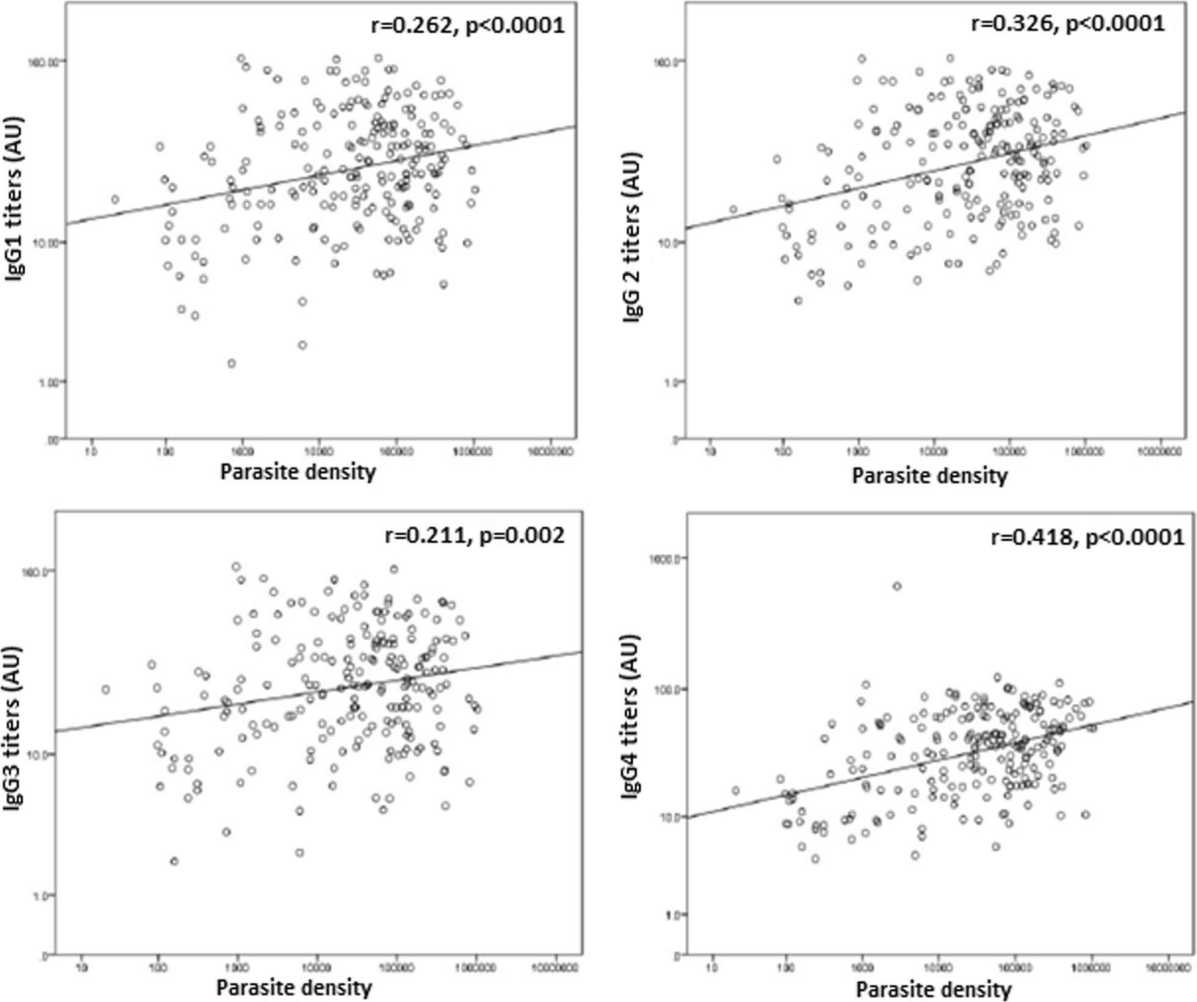
Plots of IgG subclass levels against parasite density in the study population overall. This revealed significant positive correlations between all IgG subclass levels and parasite densities in the study population

Furthermore, a significant positive correlation was observed between IgG1, IgG2, IgG3, and IgG4 titres and parasite density overall (Fig. 5). Site-specific analysis revealed a significant positive correlation between parasite density and IgG2 or IgG4 titres in Bamenda, a significant positive correlation between parasite density and IgG1 or IgG2 or IgG4 titres in Ngaoundere, and a significant positive correlation between IgG4 titres and parasite density in Limbe. No significant correlations were observed between all the IgG subclass levels and parasite density in Yaoundé and Maroua (Additional file 2).

## Discussion

Studies reporting natural immune responses against malaria parasites in children from different geographical settings in malaria-endemic areas are not readily available. Although Cameroon has all the bioecological strata of malaria present in Africa, this does not help either, as only one study has been conducted to assess the immune responses against malaria parasites in children from these different geographical zones [31]. So far no published study has evaluated the IgG subclass responses in children from the different bioecological strata in Cameroon. This study was designed to measure and compare the humoral immune responses (total IgG and IgG1–4) against MSP-1_19_ in children from five contrasting bioecological zones in Cameroon.

In this study, MSP-1 was used to evaluate the immune responses against malaria parasites because it has been shown to have a strong association with protection against clinical malaria [23, 37]. MSP-1_19_ has been reported to be conserved across distantly related *Plasmodium* species, moreover, its sequence is not constrained by function [38]. MSP-1 from *P. falciparum* was used since it is the predominant species in Cameroon [6]. This is also evident from the finding of only *P. falciparum* as the cause of clinical malaria in the target population. In this study, the levels of all the IgG subclasses increased with age overall, which corroborates the findings of other studies performed in Cameroon [39, 40]. This could be attributed to the increasing number of exposure to malaria parasite infection with age. This is further supported by the observation that clinical malaria prevalence increases with age and peaked in children between 5 and 9 years. The finding of higher prevalence of clinical malaria in this age group is in conformity with other studies conducted in Cameroon [6, 41, 42]. Furthermore, in this study, clinical malaria prevalence was significantly associated with study site, being highest in Limbe (in the C strata) and lowest in Maroua (in the SS strata). Although Maroua has an altitude that is comparable to the altitude of Limbe, the area is characterized by generally low rainfall, sparse vegetation and higher temperatures, which are unfavourable for the breeding of mosquitoes that transmit malaria. Although *An. gambiae* is the most widespread vector of malaria in Cameroon [43], its relative proportion among Culicidae in Maroua is low (11.55%) [44], which may also account for the low transmission of malaria in the area.

However, the increase in antibody response with age was not observed in all study sites. Total IgG and IgG1–4 titres against MSP-1_19_ increased with age in Bamenda (in the high western plateau strata), Limbe (in the coastal strata) and Yaoundé (in the south Cameroonian equatorial forest strata), but not in Ngaoundere (in the high inland plateau strata) and Maroua (in the sudano-sahelian strata). In Ngaoundere, a generally flattened trend was observed for all the IgG subclasses while in Maroua, total IgG, IgG3, and IgG4 did not increase with age as well. This is contrary to numerous reports that immunity to malaria increases in an age-dependent manner [45, 46]. These finding could be attributed to the low transmission intensity of malaria in the HIP and SS strata and is suggestive that adults, like children in these areas, are equally at risk of a severe malaria attack. These findings may have significant implications in the way malaria immunity is generally viewed. Further studies are required in these areas to shed more light. In the current study, there were no significant differences in total IgG and IgG1–4 levels between males and females overall, suggesting that the acquisition of malaria immunity is not dependent on gender.

The immune responses against malaria were also observed to be significantly associated with the geographical area. Total IgG and IgG1–4 antibody titres were highest in Limbe and decreased steadily northward to Ngaoundere and Maroua. Total IgG, IgG1 and IgG2 were lowest in Ngaoundere meanwhile IgG3 and IgG4 were lowest in Maroua. These discrepancies could be attributed to differences in the transmission intensity of malaria in the different ecological strata. Malaria transmission could be described as hyperendemic in Limbe, holoendemic in the Yaoundé, mesoendemic in Bamenda and Ngaoundere, and seasonal and hypoendemic in Maroua [3, 6]. The variation in the transmission of malaria in these areas is further supported by entomological data collected during the rainy season, which also showed that the entomological inoculation rate, as mentioned above, was highest in Limbe and lowest in Maroua. As evident from the current study, the prevalence of clinical malaria decreased steadily from the coastal strata (in the South) to the SS strata (in the North of the country). This may also account for the decreasing trend in the immune responses from the South to the North of the country, which is in line with previous findings [31]. The association between the immune responses against malaria and transmission intensity observed in this study is consistent with studies performed elsewhere [47–49]. The decreasing trend in the magnitude of antibody responses northward as observed in the current study may account for the increased risk of severe malaria attack and malaria mortality in the northern regions of Cameroon.

The cytophilic antibodies (IgG1 and IgG3) were consistently higher than the non-cytophilic antibodies (IgG2 and IgG4) in all the study sites except in Maroua and Ngaoundere. The findings of generally higher titres of IgG1 and IgG3 in this study is in conformity with other studies performed in Cameroon [50, 51] and elsewhere [52]. IgG1 and IgG3 are known to be protective against clinical malaria [53, 54]. These antibodies are believed to neutralize parasites directly by inhibiting the parasite, or indirectly by opsonization [55, 56].

In this study, total IgG levels were higher in children that were positive for clinical malaria compared to negative children overall. This demonstrates that infection might lead to the boosting of antibody levels, resolved infection likely leads to reduced antibody levels due to decay. This is in conformity with other studies [56, 57]. The total IgG antibody levels were higher in children positive for clinical malaria in all the study sites with the exception of Maroua where no significant difference was observed in the total IgG levels between the two groups. This could be attributed to the generally low prevalence of malaria, hence low malaria immunity in the area. Furthermore, this may also be due to the recombinant antigen used in the current study (MSP-1_19_). Studies using other recombinant antigens will be required to confirm this observation. However, site-specific analysis of the different IgG subclasses revealed no significant difference between children positive and negative for clinical malaria for IgG1 and IgG3 in Bamenda (in the HWP strata) and Limbe (in the C strata), and for all the IgG subclasses in Maroua (in the SS strata), suggesting that infection is required to mount an effective immune response against the malaria parasite. Furthermore, a significant positive correlation was observed between malaria parasite density and IgG2 or IgG4 in Bamenda, Ngaoundere and Limbe, suggestive of their role in the pathogenesis of malaria. The non-cytophilic antibodies especially IgG4 are known to be pathogenic, and their presence correlates with the severity of malaria [15, 18, 19].

This study which clearly demonstrates the variation in the immune responses against malaria parasites in children from different geographical areas in Cameroon is however limited in that participants were enrolled only during the rainy season during which malaria transmission is generally higher. Studies enrolling children during the rainy and dry seasons will be required to give a clearer picture. Furthermore, only one recombinant antigen (MSP-1_19_) was used to evaluate the antibody responses in the current study, which does not necessarily provide a complete picture of the immune responses against malaria in children in the different study sites. Further research using more recombinant antigens will be required in the study area to give a clearer picture. In addition, longitudinal studies will be required in the different bioecological strata to identify the antibodies which are protective against malaria.

## Conclusion

This study demonstrates considerable variation in the immune responses against malaria parasites in children from the different bioecological strata in Cameroon. The immune responses, which could be linked to the transmission intensity of malaria in an area, was highest in the coastal strata (where malaria transmission was highest), and lowest in the high inland plateau and sudano-sahelian strata (where malaria transmission was lowest). The immune responses were observed to increase with age in all study sites except in the sudano-sahelian (where only IgG1 and IgG2 levels increased with age) and high inland plateau (where none of the antibodies levels increased with age) strata. Furthermore, the cytophilic antibodies (IgG1 and IgG3) were consistently higher than the non-cytophilic (IgG2 and IgG4) antibodies in all the study sites except in the sudano-sahelian and high inland plateau strata. No significant differences were observed in all the antibodies levels between children positive and negative for clinical malaria in the sudano-sahelian strata. These findings may have significant implications for the design of trials for testing of malaria vaccine candidates in the country. However further research is required to identify the protective antibodies against malaria in the different ecological strata.

## Additional files

**Additional file 1: Figure S1.** Variation of total IgG, IgG1-4 antibody levels to MSP-1_19_ with age in the different study site. The line shows the LOESS smoothed estimate of the geometric mean.

**Additional file 2: Figure S1.** Plots of IgG subclass levels against malaria parasite density in Bamenda. These revealed significant positive correlations between IgG2 and IgG4 and parasite density in the study population. **Figure S2.** Plot of IgG subclass levels against malaria parasite density in Yaounde. These revealed no correlations between IgG subclasses and parasite density in the study population. **Figure S3.** Plot of IgG subclass levels against malaria parasite density in Ngaoundere. These revealed significant positive correlations between IgG1, IgG2 and IgG4 and parasite density in the study population. **Figure S4.** Plot of IgG subclass levels against malaria parasite density in Maroua. These revealed no significant positive correlations between IgG subclasses and parasite density in the study population. **Figure S5.** Plots of IgG subclass levels against malaria parasite density in Limbe. These revealed significant positive correlations between IgG4 and parasite density in the study population.

## Abbreviations

MSP: merozoite surface protein
IgG: immunoglobulin G
SCEF: south Cameroonian equatorial forest
SS: sudano-sahelian
HIP: high inland plateau
HWP: high western plateau
C: coastal
SSA: sub-Saharan Africa
ADRB: antibody-dependent respiratory burst
EIR: entomological inoculation rate
