# Supplementary material for: Comparative analysis of IgG and IgG subclasses against Plasmodium falciparum MSP-119 in children from five contrasting bioecological zones of Cameroon
Source: Malar J. 2019 Jan 22;18:16. doi: 10.1186/s12936-019-2654-9 (PMC6341684; doi:10.1186/s12936-019-2654-9)
Supplement: Supplementary file 1 — Additional file 1: Figure S1. Variation of total IgG, IgG1-4 antibody levels to MSP-119 with age in the different study site. The line shows the LOESS smoothed estimate of the geometric mean. [file 12936_2019_2654_MOESM1_ESM.docx]

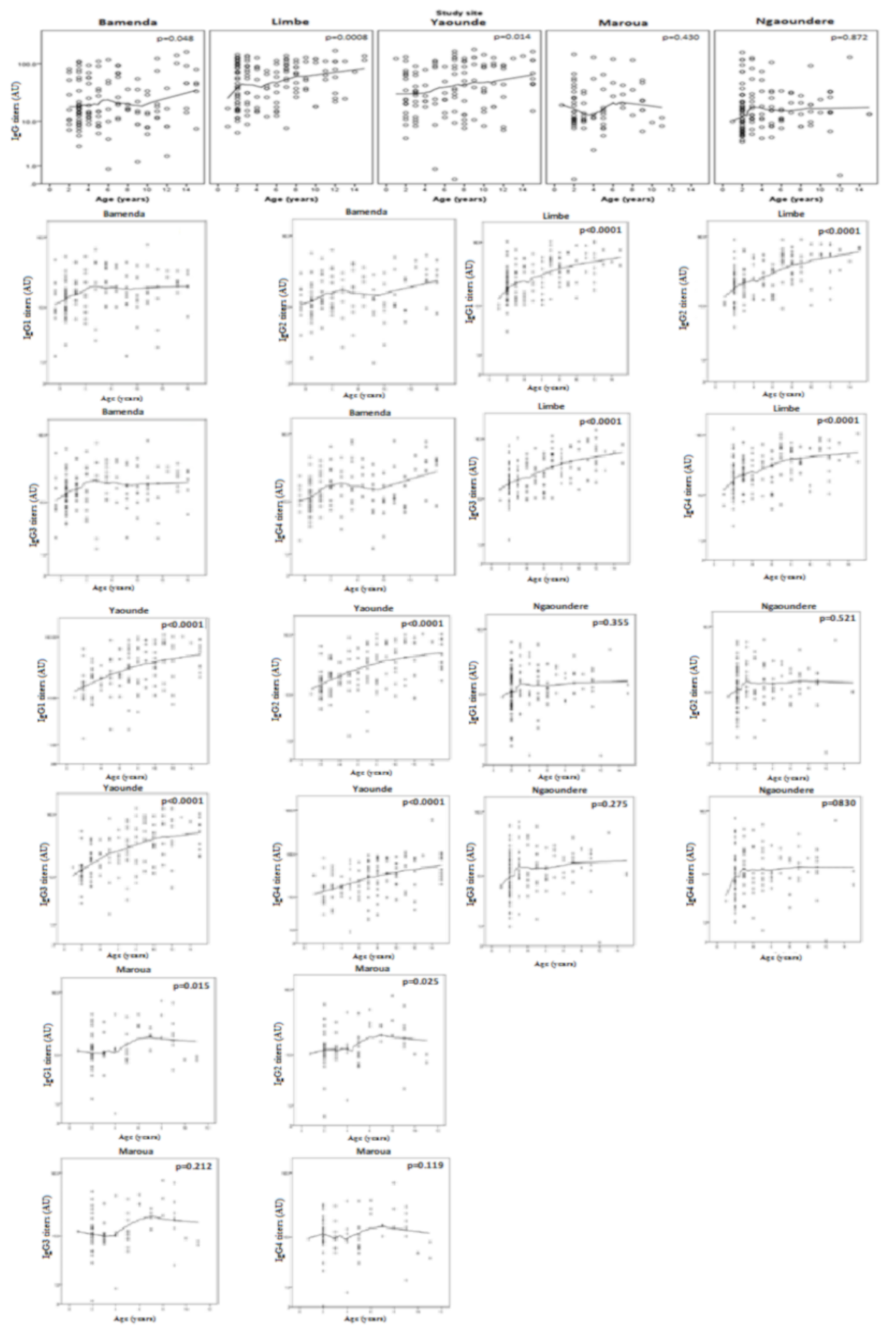


**Figure S1: Variation of total IgG, IgG1-4 antibody levels to MSP-1_19_ with age in the different study site.** The line shows the LOESS smoothed estimate of the geometric mean.
