# Supplementary material for: Comparative analysis of IgG and IgG subclasses against Plasmodium falciparum MSP-119 in children from five contrasting bioecological zones of Cameroon
Source: Malar J. 2019 Jan 22;18:16. doi: 10.1186/s12936-019-2654-9 (PMC6341684; doi:10.1186/s12936-019-2654-9)
Supplement: Supplementary file 2 — Additional file 2: Figure S1. Plots of IgG subclass levels against malaria parasite density in Bamenda. These revealed significant positive correlations between IgG2 and IgG4 and parasite density in the study population. Figure S2. Plot of IgG subclass levels against malaria parasite density in Yaounde. These revealed no correlations between IgG subclasses and parasite density in the study population. Figure S3. Plot of IgG subclass levels against malaria parasite density in Ngaoundere. These revealed significant positive correlations between IgG1, IgG2 and IgG4 and parasite density in the study population. Figure S4. Plot of IgG subclass levels against malaria parasite density in Maroua. These revealed no significant positive correlations between IgG subclasses and parasite density in the study population. Figure S5. Plots of IgG subclass levels against malaria parasite density in Limbe. These revealed significant positive correlations between IgG4 and parasite density in the study population. [file 12936_2019_2654_MOESM2_ESM.docx]

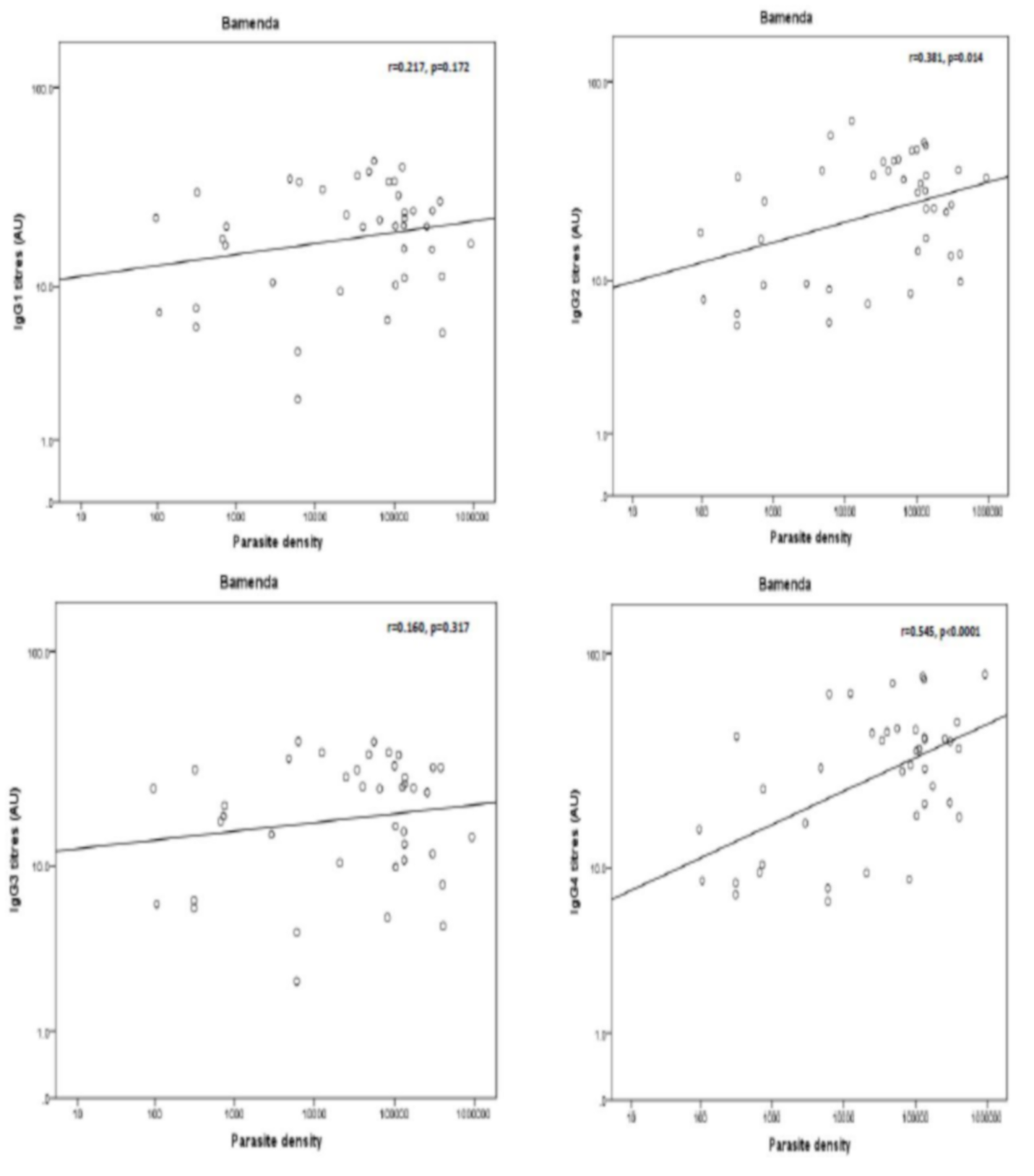


**Figure S1: Plots of IgG subclass levels against malaria parasite density in Bamenda.** These revealed significant positive correlations between IgG2 and IgG4 and parasite density in the study population.


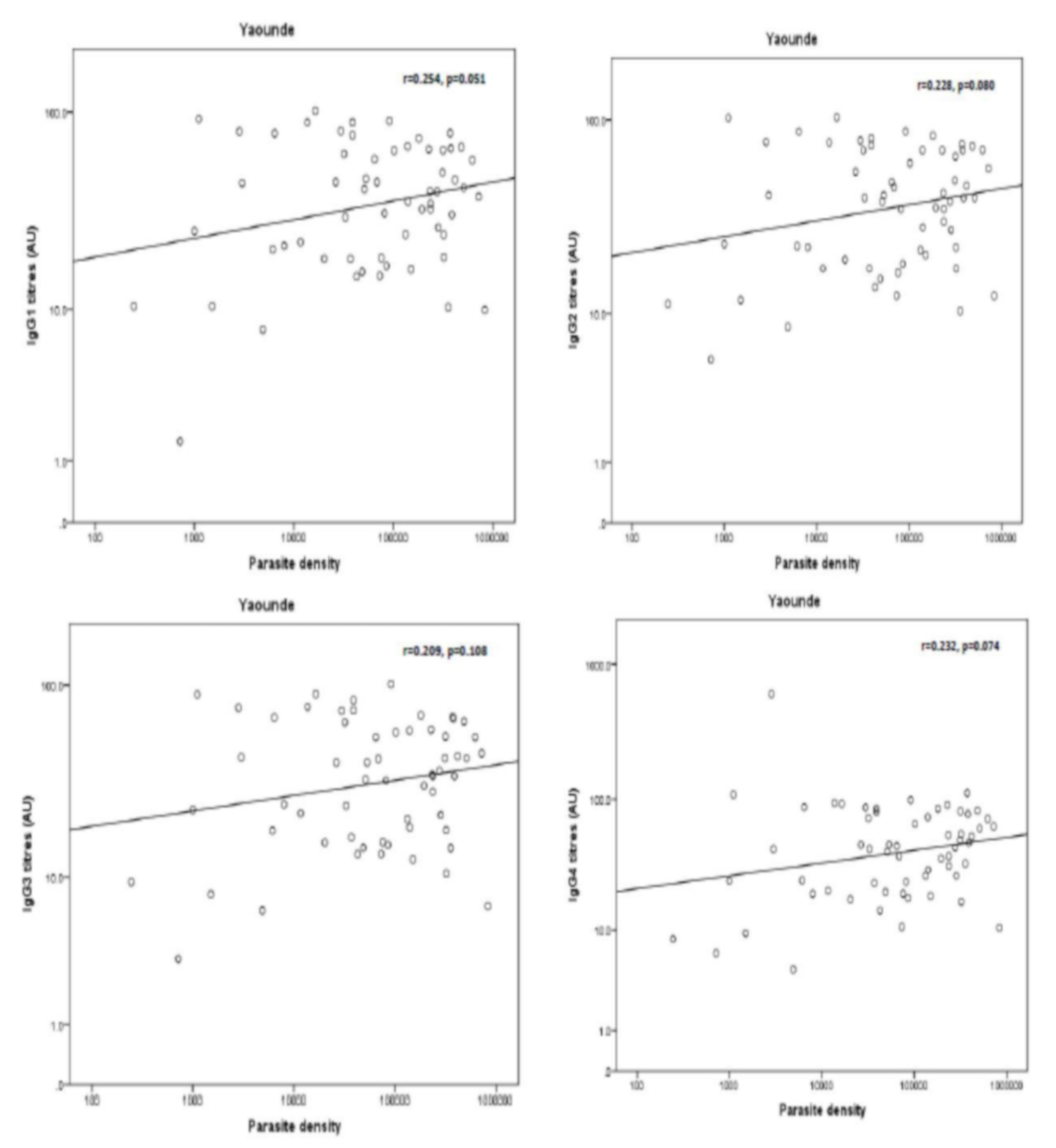


**Figure S2: Plot of IgG subclass levels against malaria parasite density in Yaounde.** These revealed no correlations between IgG subclasses and parasite density in the study population.


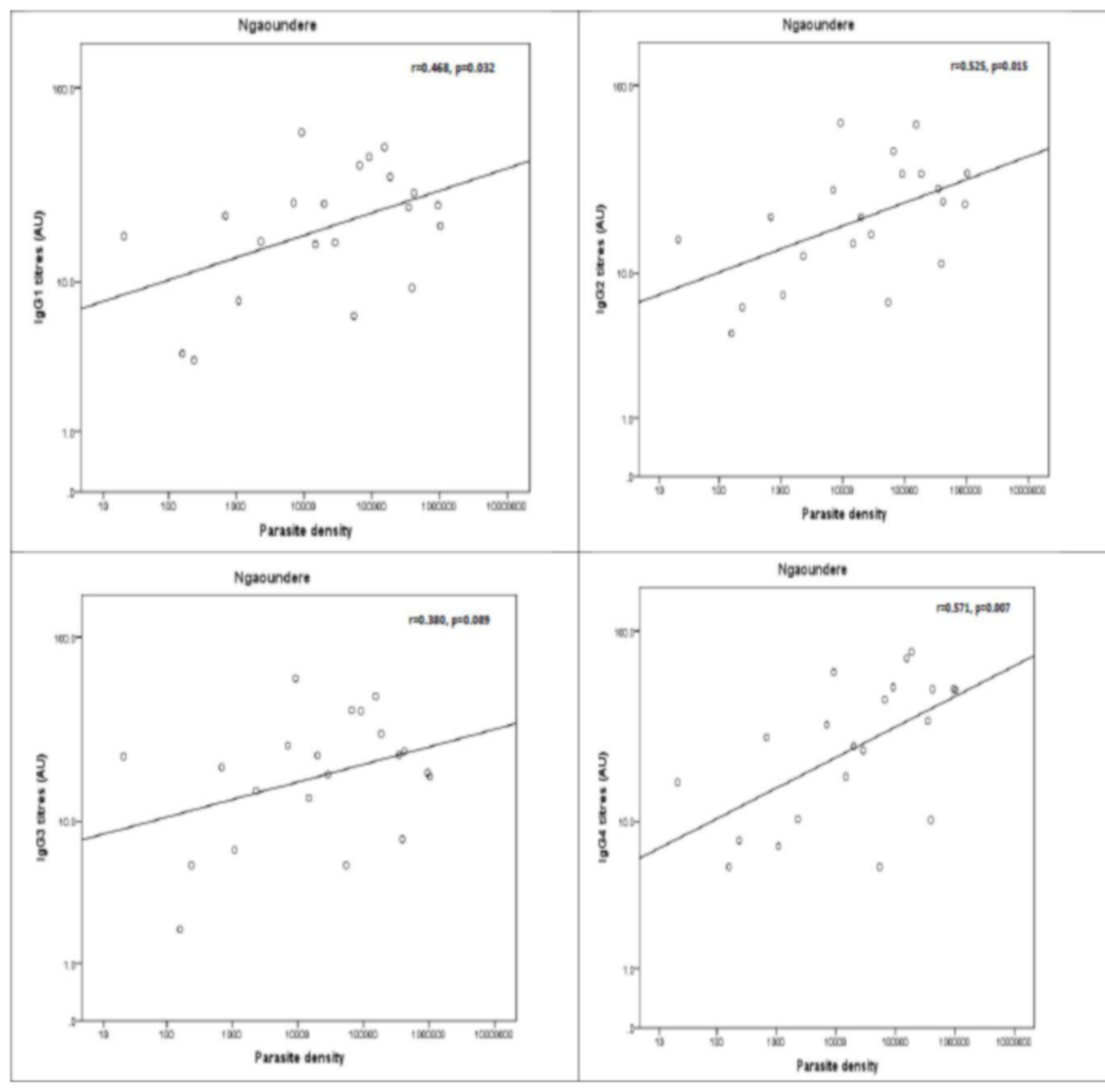


**Figure S3: Plot of IgG subclass levels against malaria parasite density in Ngaoundere.** These revealed significant positive correlations between IgG1, IgG2 and IgG4 and parasite density in the study population.


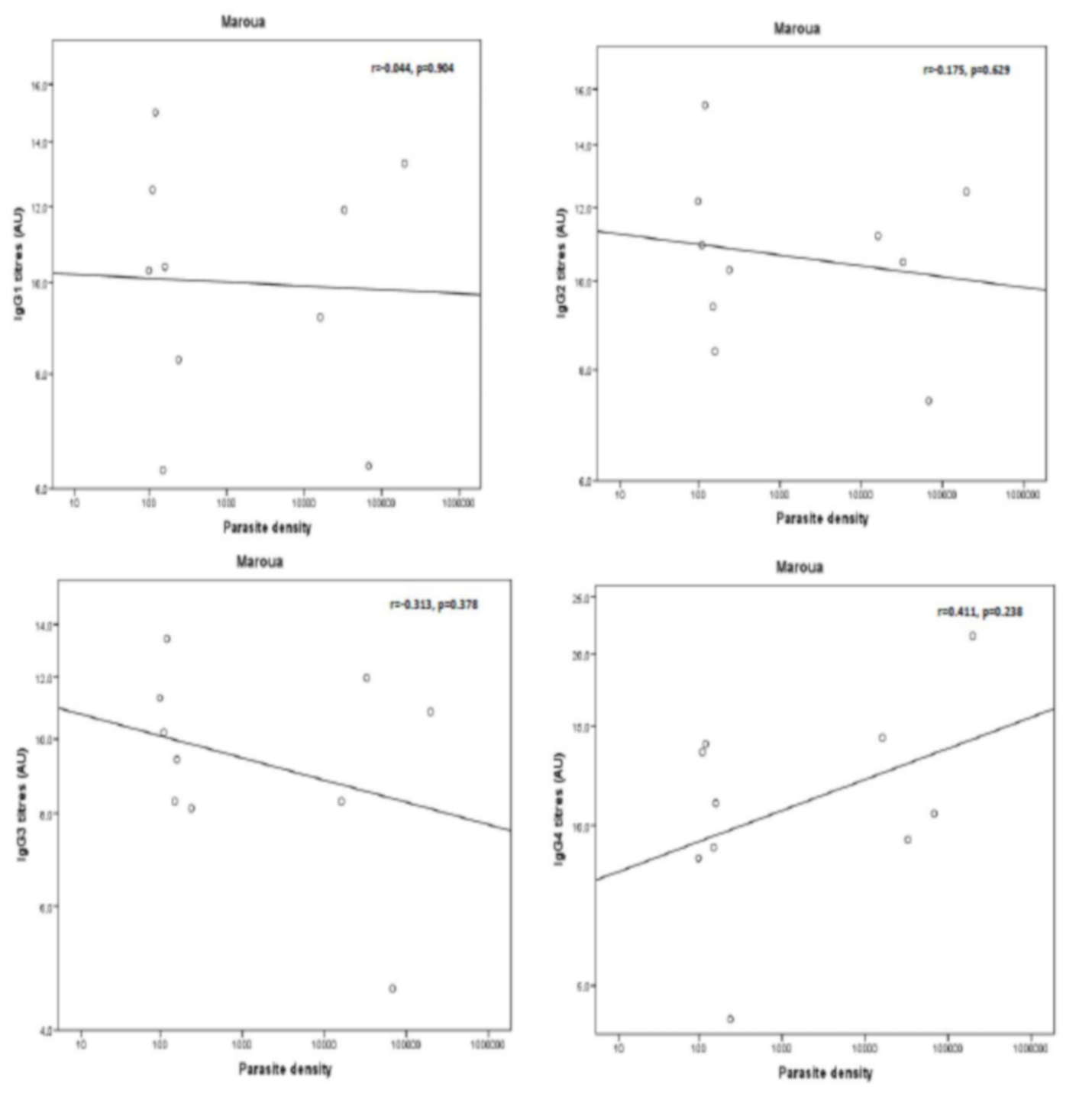


**Figure S4: Plot of IgG subclass levels against malaria parasite density in Maroua.** These revealed no significant positive correlations between IgG subclasses and parasite density in the study population.


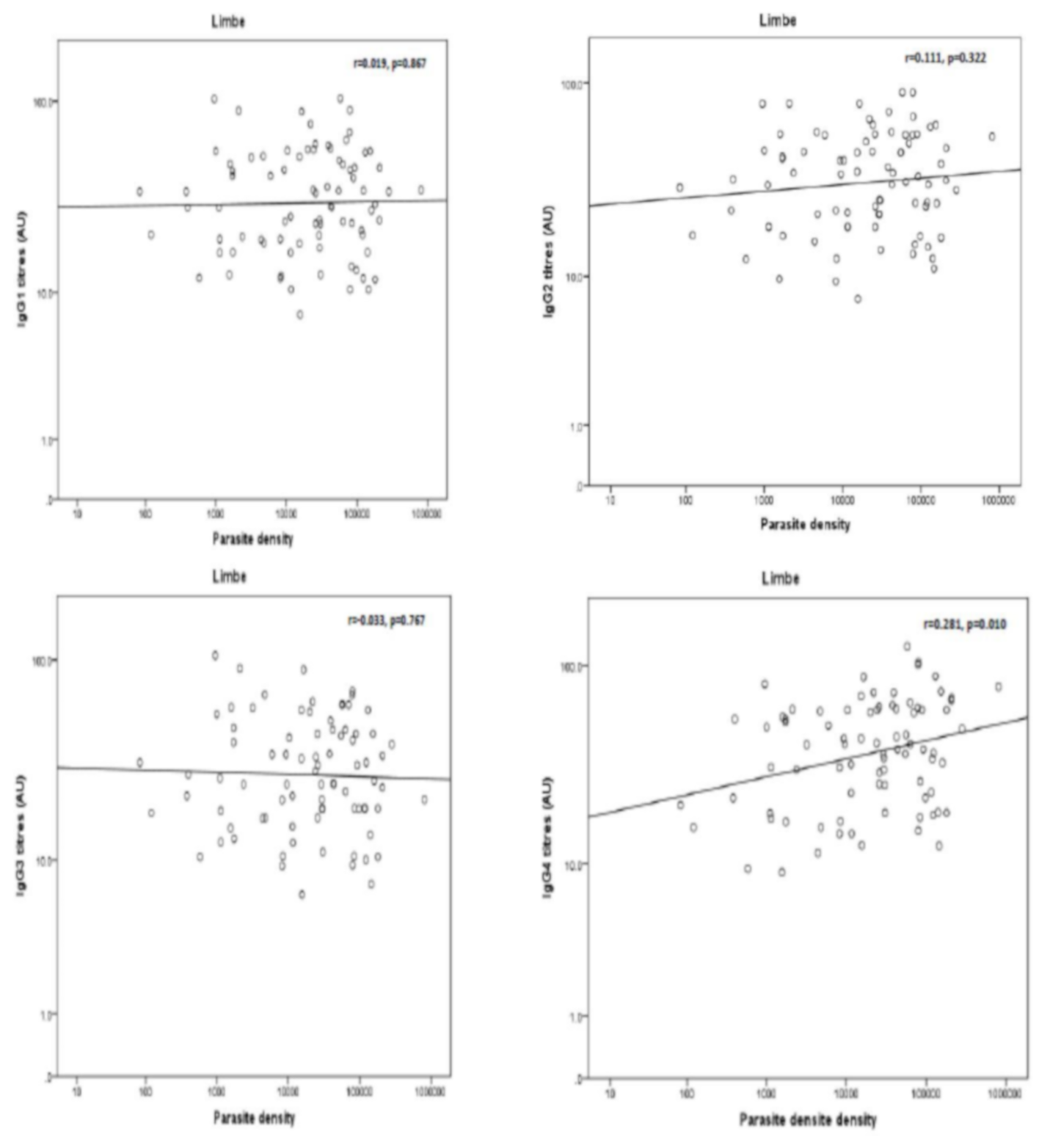


**Figure S5: Plots of IgG subclass levels against malaria parasite density in Limbe.** These revealed significant positive correlations between IgG4 and parasite density in the study population.
